# Technical and tactical evolution of the offensive team sequences in LaLiga between 2008 and 2021. Is Spanish football now a more associative game?

**DOI:** 10.5114/biolsport.2024.131818

**Published:** 2023-06-10

**Authors:** Joaquín González-Rodenas, Víctor Moreno-Pérez, Roberto López-Del Campo, Ricardo Resta, Juan Del Coso

**Affiliations:** 1 Sport Sciences Research Centre, Rey Juan Carlos University, Fuenlabrada, Spain; 2 Sports Research Center, Miguel Hernandez University of Elche, Alicante, Spain; 3 Department of competitions and Mediacoach, LaLiga, Madrid, Spain

**Keywords:** Soccer, Game style, Elite athlete, Football performance, Team sport

## Abstract

The aim of this investigation was to study the technical and tactical evolution of the offensive team sequences in the Spanish football teams from 2008/09 to 2020/21. A comparative analysis including twelve variables related to the development of offensive sequences in 4940 matches was performed from 2008/09 to 2020/21 seasons of the Spanish professional football league (LaLiga). All match observations were recorded using a validated video tracking system. Multilevel linear mixed models were used to examine the differences across seasons, considering the effects of contextual variables. The number of passes per sequence (2.4 [CI: 2.2–2.5] vs 3.2 [CI: 3.0–3.4]; +33.3%), the passing accuracy (72.1 [CI: 70.6–73.5] vs 76.9 [CI: 75.4–78.3]%; +6.8%) and the average duration of the team sequences (6.4 [CI: 5.9–6.8] vs 8.3 [CI: 7.8–8.7] seconds; +25.76%) showed a small increasing trend over the seasons (P < 0.05). In contrast, variables such as the direct speed of progression (2.2 [CI: 2.1–2.3] vs 1.6 [CI: 1.5–1.7] metres/second; -24.5%), key passes (8.1 [CI: 7.6–8.5] vs 6.8 [CI: 6.3–7.2]; -15.8%), and the sequences that ended in the attacking third (64.8 [CI: 62,7–66.8] vs 57.1 [CI: 55.1–59.2]; -11.7%) or in a shot (13.0 [CI: 12.4–13.6] vs 10.2 [CI: 9.6–10.8]; -21.6%) showed a small decreasing trend from 2008/09 to 2020/21 (P < 0.05). Spanish professional football teams slightly evolved technically and tactically towards a more associative style of play that includes longer passing sequences. This evolution also involved a decreasing speed of progression and fewer technical actions such as through balls, key passes and shots.

## INTRODUCTION

Match analysis in football (soccer) has exponentially grown in the last decades [1] and currently football is one of the most scrutinized team sports. The development of multiple technological systems (GPS devices, multi-camera tracking systems, etc.) has led to the collection of a high quantity of data during football matches even in real time [2]. This has allowed researchers and practitioners to have more opportunities to evaluate the activity of the players in relation to the technical, tactical, physical, and psychological match performance more accurately [3]. In this developing context, recent investigations have explored the technical, tactical and physical evolution of professional football competitions such as the Spanish *LaLiga* [4, 5], the English Premier League [6, 7], the German *Bundesliga* [8] and the UEFA Champions League [9]. Mainly, these investigations have been focused on the analysis of the evolution of players’ performance indicators such as the distance covered at different speeds and the number of technical actions per match. Considering that some differences can be found among competitions [10, 11], these studies revealed that high-speed running, the number of sprints and the number of passes seem to be increasing, while the total distance covered, the number of shots, crosses, tackles and clearances are showing a progressive descending trend over the seasons. In summary, these findings suggest that teams of the European football leagues are currently displaying a more possession-oriented style of play with more intense physical efforts.

Despite the relevant contribution of the above-mentioned studies, their findings are mainly based on individual technical and physical parameters, while no collective indicators were considered. In light of the necessity of exploring more interactive and representative parameters in football [12], the analysis of offensive team sequences offers key information of the collective connection of players in order to build an attack, such as number of passes per sequence, speed of progression or the final outcome of the team sequence, which can capture the team technical and tactical behaviour and performance when in possession of the ball [13]. For instance, the analysis of offensive team sequences has been a key field of study since the beginning of football performance analysis [14]. Since then, a constant debate has been carried out by researchers about the convenience and offensive effectiveness of long versus short passing sequences [15, 16]. Furthermore, the analysis of passing sequences has also been explored through other techniques such as network analysis [17] and positional data [18].

However, although there has been a constant evolution of training methods [19, 20], match strategies [21] and match analysis methods [1, 22] in the last decade that have provided insightful scientific literature and practical applications, there is still limited evidence about how the game of football has evolved over the years. Thus, it seems necessary and very interesting to explore how offensive team sequences are evolving technically and tactically to explore the effects of training and competition methods. In this analysis, the evaluation of the offensive moment could capture key technical and tactical variables related to the speed of the ball when progressing to the opponent’s goal, the duration and number of skilled actions such as passes, as well as the location of ball possession and player’s movements, which are key to evaluate styles of play [23] and to achieve football success [24].

Therefore, the aim of this investigation was to study the longitudinal technical and tactical evolution of the offensive team sequences in the Spanish *LaLiga* football teams from the 2008/09 to 2020/21 seasons.

## MATERIALS AND METHODS

### Sample

The study sample consisted of 4940 match observations in 38 professional football teams that competed in the professional Spanish football league (*LaLiga*) in any of the thirteen seasons under investigation (from 2008/09 to 2020/21). During this period, data of offensive sequences for a total of 9880 sets of data were obtained (i.e., two sets of data per match, corresponding to the two teams competing). Data were obtained from *LaLiga*, which authorized the analysis of the variables included in this investigation and the publication of results with a scientific objective. In accordance with the ethical guidelines of *LaLiga*, this investigation does not include information that identifies football players.

### Procedure

This investigation is a descriptive and comparative analysis to determine the evolution of offensive team sequences in the first division of Spanish football for thirteen seasons. The technical and tactical characteristics of each offensive team sequence were collected by Mediacoach, a video tracking system that can validly assess teams’ match statistics during match play [25]. Mediacoach collects data by using information from OPTA Sportsdata and then organizes the information to facilitate the analysis of variables for professional football teams. Recent investigations have presented the analysis of several of these data such as match statistics [26] and offensive and defensive playing style variables [27]. With this system, all the team sequences produced in each *LaLiga* match are automatically recorded and analyzed. Operationally, an offensive team sequence is defined as a passage of play in possession of the ball that belongs to one team and is ended by defensive actions, stoppages in play or a shot. For each offensive team sequence, a total of twelve technical and tactical variables were analyzed to describe the general characteristics of the team sequences, as well as the passing and the offensive performance (see Table 1). On one hand, this analysis includes collective tactical variables such as number of sequences per match, sequence width, length, duration, passes per sequence, direct speed, sequences that end in the attacking third and sequences that end in a shot. On the other hand, technical variables such as number of passes per match, passing accuracy, key passes and through passes are also included in the study.

**TABLE 1:** Description and definition of the technical and tactical variables evaluated in the present study.

Dimension	Variables	Description
Characteristics of team sequences	Number of sequences	Technical variable that quantifies the total number of offensive team sequences registered per team in a match.

	Sequence width	Tactical variable that quantifies the average distance between the leftmost point and the rightmost point reached by the ball in the offensive sequence, in metres.

	Sequence length	Tactical variable that quantifies the average distance that the ball travelled forward during each sequence per team and match, in metres.

	Sequence time	Tactical variable that quantifies the average duration of the offensive team sequences per match, in seconds.

Passing performance	Total passes	Technical variable that quantifies the total number of passes performed per team during the match.

	Passing accuracy (%)	Technical variable that quantifies the percentage of passes that are completed over the total number of passes per match.

	Passes per sequence	Tactical variable that quantifies the average number of passes performed per team during the team offensive sequences.

	Direct speed	Tactical variable that quantifies the average distance that the ball moved towards the opponent goal line during the sequence per second, in metres per second (m/s).

Offensive performance	Through balls	Technical variable that quantifies the total number of passes that penetrated through the opposing defensive line per team during the match.

	Key passes	Technical variable that quantifies the total number of passes that allow the recipient of the ball to directly shoot at goal per team during the match.

	Sequences that end in the attacking third	Tactical variable that quantifies the total number of offensive team sequences that enter and finish in the final third of the field during the match.

	Sequences that end in a shot	Tactical variable that quantifies the total number of offensive team sequences that lead to producing a shot during the match.

### Statistical analysis

The data were transferred from Mediacoach to a.csv database which was organized in Microsoft Excel. All statistical analyses were carried out using the software IBM SPSS Statistics Version 27.0. Due to the hierarchical structure of teams’ performance in football (each team has its own tactical style), a multilevel mixed model [28] was performed to cluster the collective performance (level 2) into teams (level 1). With this organization of the data, a generalized linear model was carried out to explore the longitudinal effect of the season (fixed effects) on the different tactical variables evaluated in this study, considering the effect of the team (random effects). Thus, the “team effects” represented unobserved team characteristics that influence the collective performance and account for the non-independence of the data [29]. To consider the possible contextual effects [30], the model included as fixed effects other four contextual variables: match location (home versus away), match outcome (win versus draw vs lose), ranking of the opponent in quartiles (first, second, third and fourth) and ranking of the team in quartiles (first, second, third and fourth). The Cohen F^2^ statistic was calculated as the effect size of the fixed effects [31]. The Cohen F^2^ effect size is a measure of the proportion of variance in the outcome explained by the fixed effects included in the model. In this regard, it was considered a trivial effect at a value lower than 0.02, small at a value of 0.02, medium at a value of 0.15 and large at a value of 0.35 [32].

Graphic charts with the predicted means and confidence intervals were displayed to show the longitudinal evolution of the different tactical variables throughout the seasons according to the generalized mixed linear model. Pairwise comparisons of the estimated means were performed through Fisher’s least significant test. The significance level was set to P < 0.050.

## RESULTS

Table 2 shows the comparison of data between the 2008/2009 and 2020/2021 seasons. All variables, except for “sequence width” were different between the seasons (P < 0.05). Particularly, the number of sequences largely decreased over the seasons (P < 0.05), while the sequences length moderately decreased (P < 0.05) and the sequence time had a small increase over the years (P < 0.05).

**TABLE 2:** Comparative analysis of tactical variables between the seasons 2008/2009 (reference) and 2020/2021.

Tactical variables	Coefficient	SE	*P*	ES
Number of sequences	-19.203	0.94	< 0.001	*Large*
Sequence width	0.016	0.13	0.908	*Trivial*
Sequence length	-1.236	0.15	< 0.001	*Large*
Sequence time	1.856	0.09	< 0.001	*Small*
Total passes	67.071	4.57	< 0.001	*Small*
Passing accuracy (%)	4.839	0.35	< 0.001	*Small*
Passes per sequence	0.814	0.03	< 0.001	*Small*
Direct speed	-0.530	0.02	< 0.001	*Small*
Through balls	-3.197	0.12	< 0.001	*Small*
Key passes	-1.278	0.19	< 0.001	*Small*
Sequences that end in the attacking third	-7.614	0.81	< 0.001	*Small*
Sequences that end in a shot	-2.762	0.24	< 0.001	*Medium*

SE = standard error; P = generalized mixed linear model. ES = effect size (F^2^ statistic).

Regarding the passing performance, the total number of passes, passing accuracy and passes per sequences showed a small increase in the last years (P < 0.05), while the direct speed increased (small size effect, P < 0.05). As for the offensive indicators, the number of through balls, key passes and sequences that end in the attacking third and in a shot significantly decreased from 2008/2009 to 2020/2021.

**FIG. 1:**
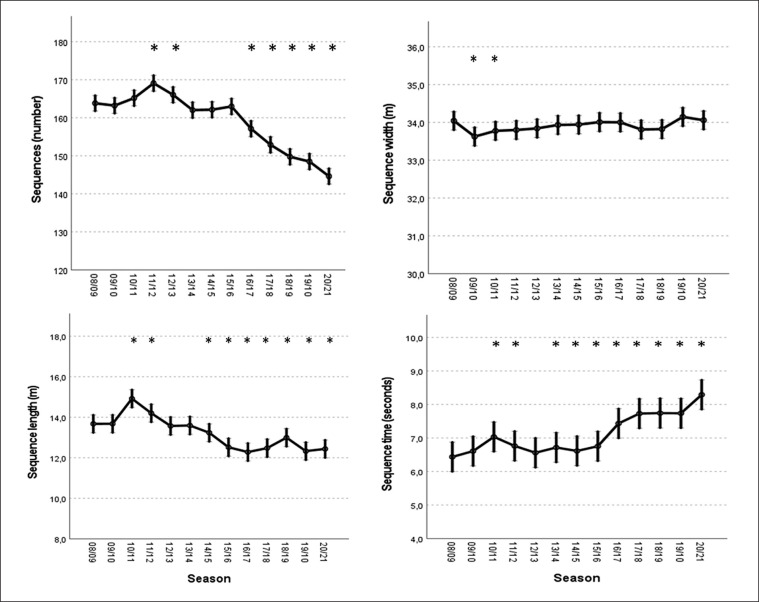
Predicted means and 95% confidence intervals for the variables “sequences”, “sequence width”, “sequence length” and “sequence time” in *LaLiga* from 2008/09 to 2020/21 according to the generalized mixed linear model. * = Significantly different from the 2008/2009 season (Fisher’s least significant test) (p < 0.05).

Figure 1 depicts the longitudinal evolution of the number of sequences per match, sequence width, length, and duration. In comparison to the 2008/09 season, the number of sequences started to decrease in the 2016/17 season (p < 0.05) and remained lower in the last five seasons under investigation. The sequence width remained stable through the seasons, rounding to the average of 34 metres, while the length of the sequences showed a descending trend over the seasons, initiating this decline in the 2014/15 season (p < 0.05).

Regarding the average duration of teams’ sequences, a significant increasing trend was found (P < 0.05), especially from the season 2014/15 onwards, when the average time of team sequences changed from 6.4 seconds to 8.3 seconds (+33.3%).

**FIG. 2:**
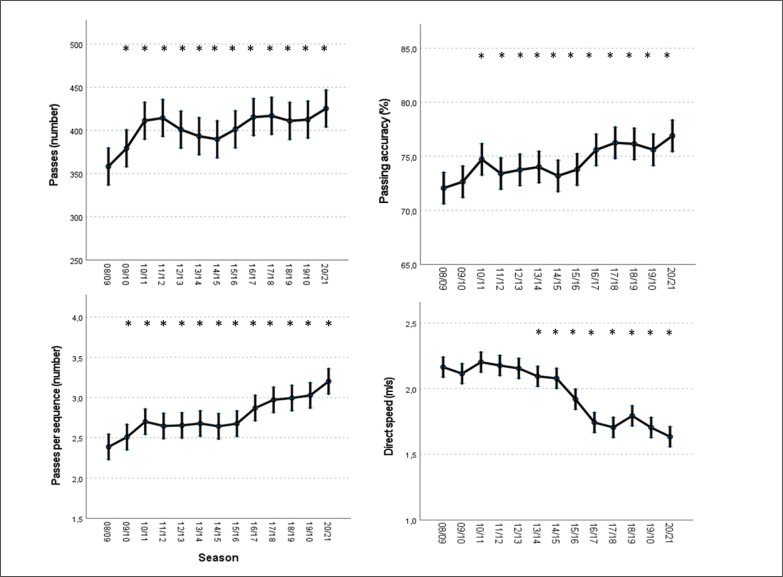
Predicted means and 95% confidence intervals for the variables “passes”, “passing accuracy”, “passes per sequence” and “direct speed” in *LaLiga* from 2008/09 to 2020/21 according to the generalized mixed linear model. *= Significantly different from the 2008/2009 season (Fisher’s least significant test) (p < 0.05).

Figure 2 shows that total passes per match (+18.7%), passing accuracy (+6.8%) and the number of passes per sequence (+34.4%) registered an increasing and constant trend over the seasons (P < 0.05). Otherwise, the direct speed of progression showed a decreasing trend, especially from the 2013/2014 season onwards (P < 0.05).

**FIG. 3:**
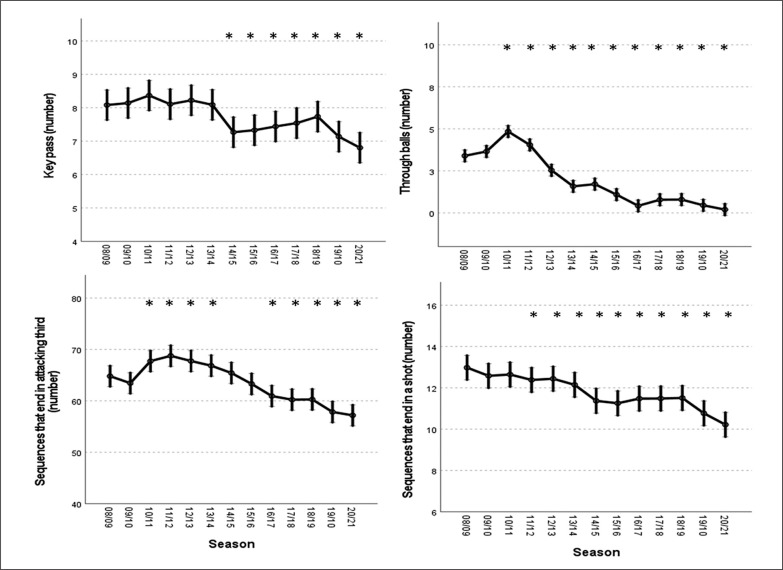
Predicted means and 95% confidence intervals for the variables “key pass”, “through balls”, “sequences that end in the attacking third” and “sequences that end in a shot” in *LaLiga* from 2008/09 to 2020/21 according to the generalized mixed linear model. *= Significantly different from the 2008/2009 season (Fisher’s least significant test) (p < 0.05).

Regarding the offensive indicators, the number of key passes significantly decreased from the 2014/15 season (P < 0.05), while the number of through balls started a decreasing process from the 2011/12 season onwards (P < 0.05). Finally, the number of sequences that ended in the attacking third gradually decreased (P < 0.05). Also, the number of sequences that ended in a shot decreased (p < 0.05) with statistically significant differences between the 2008/09 season and all the seasons after the 2011/12 season (Figure 3).

## DISCUSSION

The aim of this investigation was to study the technical and tactical evolution of the team offensive sequences in *LaLiga* football teams from the 2008/09 to the 2020/21 seasons. The main outcome of this investigation was that offensive team sequences in *LaLiga* have significantly and slightly changed towards a lower number but longer offensive sequences, including a small increase in passes, accuracy, and duration. However, the number of sequences that ended in the attacking third or ended in a shot, as well as the number of technical actions such as through balls and key balls, progressively and slightly decreased. Collectively, this information indicates that *LaLiga* football teams evolved tactically towards a more associative style of play that includes longer passing sequences at the cost of decreasing speed of progression during the game. These results also suggest that football teams now produce more elaborate sequences until the opportunity to attack is present, as the number of goals has not changed [4], despite there being a lower number of sequences that end in the attacking third and those ending in a shot.

Our study revealed that the number of passes per match, the number of passes per sequence and the average duration of each sequence showed a small increasing trend over the seasons. Although existing literature has reported that the Spanish *LaLiga* is characterized by a more combinative style of play in comparison to other leagues [10, 33], this study confirms that this style is the result of a constant evolution, at least since the 2008/09 season. In line with these results and focusing on the role of specific playing positions, Lago-Peñas et al. [5] found that, in *LaLiga*, central backs significantly increased the number of total passes and the number of long passes from 2012 to 2019. This indicates that central backs are now more involved in the build-up phase of the game and suggests that offensive sequences are now formed further from the opposing team’s goal. Additionally, the direct speed, a variable that measures the capacity of the team to progress in each sequence, as the average speed of ball movement towards the opponent’s goal line during the sequence, has decreased, suggesting that sequences are now more horizontal, reducing the use of direct play game styles.

In this regard, Yi et al. [9] recently observed that teams in European leagues are now more focused on the control of match play by increasing passing frequency and accuracy, which coincides with the outcomes of the current investigation. Other studies have also reported an increasing passing performance in English Premier League from 2006 to 2013 [7] and the German *Bundesliga* from 2014 to 2017 [8]. In contrast, Zhou et al. [34] did not find any increase in the passing performance in the Chinese Super League from 2012 to 2017. Although these results reflect the different technical and tactical development of each domestic competition according to cultural, economic, and social dimensions, it seems that there is an international tendency for a higher passing frequency in elite football teams, converting football today into a more combinative sport than it was a decade ago.

In the Spanish context, this technical and tactical evolution towards a more associative game seems to have had a small boost during the 2010/11 season. In this season, the number of passes per match increased by 8.4% from the previous season (Figure 2), with 8.0% more passes per sequence. It is interesting to mention that this season took place just after the Spanish National team won the 2010 World Cup tournament in South Africa with a very popular possession-oriented style of play [35, 36]. Also, the 2010/11 season was played two years after Pep Guardiola took over FC Barcelona in a very successful period that started by winning one Champions League title and two consecutive *LaLiga* trophies with a style of play characterized by making a great number of passes and creating constant interactions and connections between players [37]. Thus, the possible effect of these two successful teams that implemented possession-oriented styles of play may have influenced the rest of the Spanish teams to adopt more associative passing sequences in the next years to try to achieve higher success.

Despite the small increase in the number of passes and time per sequence, our study found that the length of the sequences and the speed of progression showed a small descending trend over the seasons. From a practical perspective, these two changes suggest that teams progressed a shorter distance than before, despite using longer sequences. Furthermore, the number of through balls and key passes also showed a descending trend. Although further research is needed to understand the current attributes of passing in football, these results do not indicate that passing tempo or ball speed has decreased, but that teams now decide to produce more elaborate plays with more horizontal and backward passes until a favorable moment to attack and progress is obtained. Additionally, these findings suggest that teams could have strengthened their defensive organization [4], and pressure on the ball, which would make it more difficult to penetrate through the opposing lines, forcing attacking teams to display more passing combinations to overcome the defensive teams. This tactical scenario with both a more offensive combination and defensive organization could reduce the opportunities for the teams to have moments of transitions from defense to attack to recover the ball, which is very effective to create goal-scoring opportunities [38, 39]. Similar to these results, Konefal et al. [8] suggested that the evolution of football seems to be directed towards more collective and organized tactics, reflecting a better understanding of the tactical roles of players. These technical and tactical features could have a physical impact so that this better collective organization could help players to optimize their physical efforts and save energy to perform higher-intensity actions during the game, which is another new feature of modern football [40]. In line with this interpretation, the recent study by Lago-Peñas et al. [5] recorded a reduction in the total distance covered by *LaLiga* players, while the number of efforts made at high-intensity running increased in recent seasons.

As for the offensive performance, our data revealed that those sequences that ended in the attacking third or achieved a shot showed a descending trend over the seasons. Our results support the findings of Errekagorri et al. [4], which demonstrated how Spanish teams produced a smaller number of technical actions such as shots and crosses in recent seasons. These facts are surely aligned with the previous findings that indicated fewer through balls and key passes, which is probably due to the better protection of key spaces by the defensive teams. These technical and tactical characteristics seem to create a more controlled and predictable offensive context where penetrating into the final third of the pitch or achieving a shot could become more difficult than several years ago.

This study is not exempt from limitations. Firstly, it is crucial to mention that this investigation was performed following a static approach [41], where data are collected per match without considering the contextual variables that change throughout the match. Additionally, the current study was carried out with data from a national football league of elite male players and the results of the investigation should not be extrapolated to other leagues, other categories, or to women’s football.

## CONCLUSIONS

The current data show that football teams competing in *LaLiga* have slightly evolved technically and tactically towards a more associative and combinative style of play that includes longer passing sequences both in time and quantity of passes. This technical and tactical evolution also presents a decreasing speed of progression and distance progressed in each sequence, as well as a small decrease in the number of through balls, key passes, and shots.

As practical applications for football coaches and sporting directors, it seems that football tactics are in a slight evolution towards a more associative style of play with less offensive verticality and penetration, which requires coaches not only to train players to be accurate in their actions to possess the ball but also to be able to acquire excellent dribbling or passing skills to disrupt the defensive organization of the opposing team. Additionally, as the number of through balls, key passes, and shots has been decreasing in recent years, having players with attributes to produce these actions may be key to having a successful football squad. The knowledge provided by this study encourages football coaches and analysts to reflect on the actual development of football tactics.

## Data Availability

The data that support the findings of this study are available from *LaLiga*. Restrictions apply to the availability of these data, which were used under license for this study. Data are available from the authors with the permission of *LaLiga*.
